# Selection of a Probiotic for Its Potential for Developing a Synbiotic Peach and Grape Juice

**DOI:** 10.3390/foods13020350

**Published:** 2024-01-22

**Authors:** Virginia Prieto-Santiago, Ingrid Aguiló-Aguayo, Jordi Ortiz-Solà, Marina Anguera, Maribel Abadias

**Affiliations:** Institute of Agrifood Research and Technology (IRTA), Postharvest Program, Edifici Fruitcentre, Parc Agrobiotech Lleida, Parc de Gardeny, 25003 Lleida, Spain; virginia.prieto@irta.cat (V.P.-S.); ingrid.aguilo@irta.cat (I.A.-A.); jordi.ortiz@irta.cat (J.O.-S.); marina.anguera@irta.cat (M.A.)

**Keywords:** probiotic, prebiotic, synbiotic, viability, antimicrobial

## Abstract

Due to recent interest in the potential of probiotics as health promoters and the impact of health and environmental concerns on eating habits, non-dairy probiotic food products are required. This study aimed to evaluate the viability of different probiotic microorganisms in peach and grape juice (PGJ) with or without the prebiotic inulin and their antimicrobial activity against the foodborne pathogen *Listeria monocytogenes* and the juice spoilage microorganism *Saccharomyces cerevisiae*. Firstly, the viability of seven probiotic strains was studied in PGJ with an initial concentration of 10^7^ CFU/mL for 21 days at 4 °C and for 3 days at 37 °C. In parallel, the physicochemical effect, the antimicrobial effect and the lactic acid production in PGJ were evaluated. Secondly, the probiotic with the best viability results was selected to study its antimicrobial effect against *L. monocytogenes* and *S. cerevisiae*, as well as ethanol and acetaldehyde production by the latter. *L. casei* showed the highest viability and grew in both refrigerated and fermentation conditions (1 log), produced the greatest lactic acid (5.12 g/L) and demonstrated in vitro anti-*Listeria* activity. Although the addition of the prebiotic did not improve the viability, lactic acid production or anti-*Listeria* activity of the probiotics, under the conditions studied, the prebiotic potential of inulin, support the design of a synbiotic juice. Finally, although none of the probiotic, fermentation products, or postbiotics showed any antimicrobial activity against *L. monocytogenes* or *S. cerevisiae*, the addition of *L. casei* to the PGJ significantly reduced the production of *S. cerevisiae* metabolite ethanol (29%) and acetaldehyde (50%). *L. casei* might be a suitable probiotic to deliver a safe and functional PGJ, although further research should be carried out to determine the effect of the probiotic and fermentation on the nutritional profile of PGJ.

## 1. Introduction

Today’s lifestyle changes are shifting eating patterns towards plant-based diets. This shift is driven not only by the well-known health benefits but also by the lower environmental footprint of these diets. In addition, the growing demand for healthier, nutrient-rich and functional foods and the demonstrated impact of diet on the risk of various disorders have focused the current dietary pattern on the consumption of health-enhancing foods [1].

In addition, compared to the well-established functional ingredients such as vitamins and minerals, pro- and prebiotics have been intensively studied in the last decade, due to their potential beneficial effects associated with the maintenance of an adequate microbiota balance and subsequent health promotion [2]. The Panel of the International Scientific Association for Probiotics and Prebiotics (ISAPP) [3] defined probiotics as “live microorganisms that, when administered in adequate amounts, confer a health benefit on the host”. Lactic acid bacteria (LAB) are commonly used as probiotics, and of these, *Lactobacillus* and *Bifidobacterium* genera are mainly associated with spontaneous food fermentation and health benefits [4]. Nevertheless, the increasing demand of ready-to-eat food and the fact that vegetative forms, such as LAB species, are susceptible to processing conditions like heat treatments, more suitable species that present spore forms, such as *Bacillus*, are being studied [5].

Current knowledge associates the beneficial potential of probiotics with an increased presence of these microorganisms to the detriment of potential pathogenic bacteria in the gut. It has been demonstrated that it is possible to temporarily alter the composition of the gut microbiota of healthy individuals in favor of the above-mentioned bacterial species, after ingestion of some probiotic-containing foods [6]. Although it is not clear whether the alteration of the microbiota is a cause or a consequence of a physiopathology, it is scientifically reported that a balanced microbiota is of great importance for proper health maintenance [2]. Several strains of *Lactobacillus* and *Bifidobacteria* have been applied as supplements for the treatment of diarrhea (e.g., *Lactobacillus acidophilus* LB) [2,7] and in food products for the promotion of intestinal health and disease prevention (e.g., *Lactobacillus acidophilus* L-92, *L. casei*) [8]. Probiotics are not only recognized due to their beneficial gastrointestinal effects but also because of their ability to modulate the immune response. Indeed, several positive effects have been reported in acute rotavirus diarrhea or atopic dermatitis, after the intake of *Lactobacillus rhamnosus* GG [9] and a mixture of *Bifidobacterium animalis* subsp. *lactis* CECT 8145, *Bifidobacterium longum* CECT 7347 and *Lacticaseibacillus casei* CECT 9104 [10,11], respectively. Not only LAB but also *Bacillus* present some health potential, related to the prevention of pathologies in the gastrointestinal tract [12,13] and respiratory conditions [14].

But the benefits of probiotics may go beyond promoting human health; their antimicrobial potential may be an exploitable aspect in the food industry. In fact, the increase in the number of foodborne disease outbreaks could be explained by the growing demand for prepared and minimally processed foods, which require a longer shelf life and are particularly vulnerable to contamination with foodborne pathogens [15]. Therefore, the development of new methods for efficient and low-cost preservation without the use of chemical additives, while maintaining taste, texture, sensory and nutritional properties is of considerable interest [16]. Thus, one of the alternatives being explored is biopreservation, either using microorganisms or their metabolites [17]. Protective cultures can potentially control the contamination and spread of undesirable microorganisms by occupying the same niche as them (competing for space and nutrients) and/or by producing antimicrobial substances. Specifically, probiotics are able to prevent pathogens from attaching enterocytes and activate an immune response against pathogens [18]. Lactic acid bacteria can be used as bioprotective cultures [19]. Another method of biopreservation is the application of metabolites produced by the cultures, including antimicrobial peptides such as bacteriocins [20,21]. *Bacillus coagulans* along with LAB and their bacteriocins have been reported to present an antimicrobial effect [22,23,24].

The antimicrobial effect of probiotics has already been reported against several food-borne pathogens, such as *L. monocytogenes* in different food matrices [25]. Previous work has demonstrated that several *Lactobacillus* strains might present the potential to inhibit *L. monocytogenes* when applied in food, such as fruit [26,27]. However, the most common contamination in food, particularly in beverages, is related to spoilage microorganisms that deliver metabolites with unpleasant characteristics. *Saccharomyces* spp. is one of the most frequent spoilage microorganisms found, typically, in sweet beverages. It takes advantage of the sugar compounds present in the matrix to survive and, as a result, releases unpalatable compounds into the food matrix. Although BAL are not usually applied to prevent or inhibit contamination from *S. cerevisiae,* or another spoilage microorganism, antifungal activity has been described for *Bacillus* isolates [28].

The most common method to provide probiotic microorganisms is through fermentation. Fermentation is the oldest method of food preservation and, typically, is generated spontaneously in plant material, resulting in changes in the nutritional, physicochemical and sensory profile of the food product [29]. The microorganisms involved in the fermentation process are mainly LAB, and dairy products are the most consumed fermented products. Scientific research has attributed this to fermented food health properties, such as prevention cardiovascular and immune-related diseases. Some of the functional compounds produced in this process are lactic, acetic and propionic acids, vitamins (B12) and essential amino acids [29,30].

In order to provide probiotics, it is also possible to add them in a food matrix, but without fermentation (‘probiotication’) [31]. In this regard, probiotic microorganisms are usually found in both fermented and non-fermented dairy products; nevertheless, increasing lactose intolerance prevalence and alternative food patterns, such as veganism, along with the health concern related to diet, non-dairy products, such as fruits, vegetables, cereals, etc., are being explored as suitable carriers of probiotics [32].

Fruit juices are obtained from the edible parts of fruit. Due to their nutrient-rich composition, fruit juices are susceptible to fermentation but not typically fermented [33]. Moreover, fruit juices are a great source of antioxidants, vitamins, minerals, bioactive compounds and are mostly accepted and appealing for most of the population, and they are considered to be good carriers of probiotic microorganisms. Several studies have already described the benefits, not only after the incorporation of probiotics but also through a fermentation process, in fruit juices [34]. However, it is also reported that the low pH and high concentration of organic acids present in most fruit juices negatively affect the viability of probiotics [35].

Not only are probiotics interesting for health promotion, but prebiotics also play an important role as elementary components of healthy dietary patterns. Prebiotics are dietary substrates that can be used by beneficial microorganisms (probiotics) placed in the gastrointestinal tract [36]. These compounds can potentially promote microbiota balance by enhancing beneficial bacteria and decreasing, consequently, pathogenic bacteria. Some beneficial aspects derived from the consumption of prebiotics are related not only to the promotion of probiotic microorganisms but also to the ones typically related to fiber, to which prebiotics generally belong. Fructooligosaccharides (FOS) are described as prebiotics, due to their action in stimulating the growth of beneficial *Bifidobacteria* in the gut and their positive impact on human health by reducing the total cholesterol and serum lipids and preventing constipation, among other benefits [37]. Inulin is a biodegradable fructoligosaccharyde, mainly extracted from crops such as roots and tubers, among others. Inulin consists mainly of a β-d-fructan unit linked (2 → 1) with a glucopyranose unit at the reducing end. This special structure of inulin prevents it from being degraded by any digestive enzyme, and consequently, it reaches the small intestine, where it is available, as a nutrient, for beneficial microorganisms. Inulin has been widely used not only in food industries as a substitute of sucrose and now as a prebiotic but also in other sectors such as animal feed, biofuel and water purification [38].

Considering the new dietary guidelines, the latest concerns related to human and environmental health and the beneficial effects of probiotic microorganisms, it would appear essential to investigate the potential of non-dairy food matrices in order to deliver probiotics.

Thus, the objective of this work was to explore the viability of seven probiotic species, their antimicrobial activity and their impact on physicochemical quality in peach and grape juice, with and without prebiotic, during refrigeration and fermentation. In addition, the antimicrobial potential of the selected probiotics was extensively studied. 

## 2. Materials and Methods

### 2.1. Material

#### 2.1.1. Juice

Commercial pasteurized peach and grape juice (PGJ), obtained from ecologically produced fresh fruits (50% of each) with no sugar added and additive free (Cal Valls, Vilanova de Bellpuig, Lleida, Spain) was used.

#### 2.1.2. Prebiotic

The effect of the addition of the prebiotic inulin (2%) was investigated. Sweet inulin (Soc Chef, SLU, Tàrrega, Lleida, Spain) was added to PGJ.

#### 2.1.3. Microorganisms

Six of the probiotics used in this study belong to *Lactobacillus* genera. *Lactobacillus rhamnosus* GG (ATCC 53103, LRGG) (from Ashtown Food research Centre; Teagasc; Ashtown, Dublin, Ireland); *L. rhamnosus* SP1 (SP1), Lyofast SYNBIO 100 (SYNBIO), which consisted of a mixture of *L. rhamnosus* IMC 501 and *Lactobacillus paracasei* IMC 502; *Lactobacillus acidophillus* LA3 (LA3); and *Lactobacillus reuteri* LR91 (LR91) were provided by Sacco System (Italy), and *Lacticaseibacillus casei* CECT 9104 (formerly *Lactobacillus casei*, LC) was isolated from the commercially available capsule Dermaveel Pro (Heel España, Madrid, Spain). One thermoresistant bacteria *Bacillus coagulans* 04 (BC04), which was obtained from Sacco System, was assessed also as a probiotic.

A cocktail of 5 *Listeria monocytogenes* strains were used in the antimicrobial tests: serovar 1a (CECT-4031), serovar 3a (CECT-933), serovar 4d (CECT-940), serovar 4b (CECT-4032) and serovar 1/2a (Lm_230/3), which was previously isolated in our laboratory from a fresh-cut lettuce sample [39]. The yeast *Saccharomyces cerevisiae* WDCM00058 was used as model of a spoilage microorganism.

### 2.2. Methodology 

#### 2.2.1. Microorganisms Preparation

**Figure 1:**
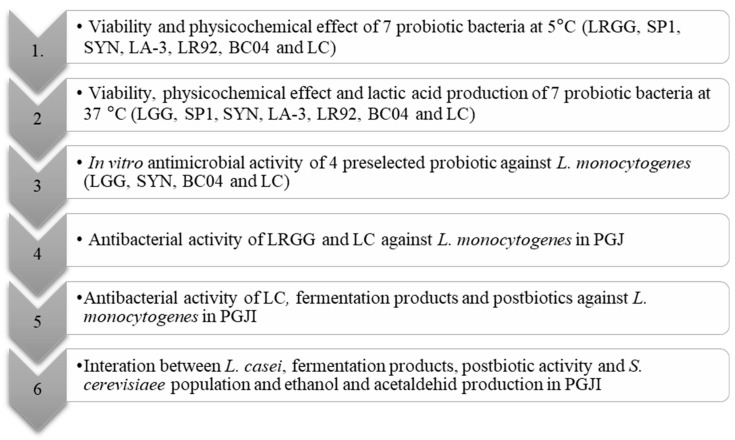
Experimental design. *Lactobacillus rhamnosus* GG (LRGG), *L. rhamnosus* SP1 (SP1), SYNBIO 100 (SYNBIO), which consisted of a mixture of *L. rhamnosus* IMC 501 and *Lactobacillus paracasei* IMC 502, *Lactobacillus acidophillus* LA3 (LA3), *Lactobacillus reuteri* LR91 (LR91), *Lactobacillus casei* CECT 9104 (LC), *Bacillus coagulans* 04 (BC04), peach and grape juice (PGJ) and peach and grape juice with 2% of inulin (PGJI).

The experimental design is carefully explained in Figure 1. The probiotic strains *L. rhamnosus* GG (LRGG) and *L. casei* (LC) were grown in de Man, Rogosa and Sharpe (MRS, Biokar Diagnostics, Beauvais, France) broth for 20–24 h at 37 ± 1 °C. The other five probiotic microorganisms, which were provided freeze-dried, were added directly in the PGJ to obtain an initial concentration of 10^7^ CFU/mL of juice.

*L. monocytogenes* strains were grown individually in tryptone soy broth (TSB, Biokar Diagnostics, Beauvais, France) supplemented with 6 g/L of yeast extract (TSBYE, Biokar Diagnostics, Beauvais, France) for 20–24 h at 37 ± 1 °C.

The yeast *S. cerevisiae* was grown on Yeast extract peptone dextrose (YPD, Biokar Diagnostics, Beauvais, France) broth (5 g/L yeast extract, 10.0 g/L peptone and 20.0 g/L glucose) at 25 ± 1 °C for 48 ± 4 h.

Yeast and bacterial cells were obtained by centrifugation at 9800× *g*, 10 min at 10 °C. The broth was decanted, and the cells were resuspended in saline solution (SS; 8.5 g/L NaCl). In the anti-*Listeria* tests, equal volumes of the five *L. monocytogenes* concentrated suspensions for each strain were mixed.

Population in concentrated suspensions of each microorganism were checked by plating appropriate dilutions onto MRS agar (for acid-lactic probiotic bacteria) and tryptone soy agar (TSA, Biokar Diagnostics, Beauvais, France) for *B. coagulans* 04, PALCAM agar (PALCAM Agar Base with selective supplement, Biokar) for *L. monocytogenes* and onto YPD agar for *S. cerevisiae*. MRS and PALCAM plates were incubated at 37 ± 1 °C for 24 and 48 h for probiotic bacteria and *L. monocytogenes*, respectively, and the YPD for *S. cerevisiae* plates were incubated at 25 ± 1 °C for 48 h.

#### 2.2.2. Survival of the Probiotics in PG Juice at Refrigeration (5 °C) and 37 °C, Fermentation Capability and Effect on the Physicochemical Properties

Probiotic strains (LRGG, SP1, SYN, LA3, LR91, BC04 and LC) were inoculated in the PGJ (with or without 2% inulin) from the concentrated suspensions, prepared as indicated above, to obtain 10^7^ CFU/mL. Afterwards, PGJ was stored at 5 °C. After inoculation and after 3, 7, 14 and 21 days of storage, the population of the studied probiotic was determined by serial decimal dilutions followed by plating on MRS medium for LAB. BC04 was plated in TSA medium and incubated at 37 °C for 48 h in high CO_2_ concentration (6.0 ± 0.5%). Plates were counted and results expressed as log CFU/mL. To study the capability of the probiotic strains studied to grow and/or ferment the juice, inoculated PGJ were incubated at 37 °C. Initially and after 24, 48 and 72 h, populations were determined as indicated for 5 °C.

On each sampling day, pH, total soluble solids (TSS) and titratable acidity (TA) were determined in triplicate. pH was determined in a pH-meter model GLP22 (Crison Instruments SA, Barcelona, Spain). TA was measured by titration of 10 mL of juice with 10 mL of distilled water with 0.1 M NaOH until pH 8.2 was reached, and results were indicated as g malic acid/L. TSS was measured with a refractometer (Atago Co., Ltd., Tokyo, Japan), and the results were expressed as °Brix. In PGJ juices incubated at 37 °C, D-lactic and L-lactic acid production were also determined in presence and in absence of inulin using the Enzymatic kit from Biosystem (Barcelona, Spain) (ref. 12801 and 12802, respectively) [40]. Briefly, after the clarification, 10 µL of the juices were placed with D- and L-lactic dehydrogenase and NAD+ (25 mmol/L) in a multiwell plate and the absorbance was collected at 340 nm, in FLUOstar Omega spectrophotometer (BMG Labtech, Ortenberg, Germany). The analysis was carried out in triplicate, and results were expressed in g total lactic acid/L.

#### 2.2.3. Antimicrobial Activity of Selected Probiotics against *L. monocytogenes* In Vitro

The disk diffusion test was performed to test in vitro antimicrobial activity of the four probiotic microorganisms selected (LC, LRGG, SYNBIO and BC04) against five strains of *L. monocytogenes* following the methodology described by Nicolau-Lapeña et al. [41]. Plates were prepared with a thin layer of TSB. Semi-solid TSBYE agar tubes (5 mL) were prepared and kept at 4–5 °C. Tubes were inoculated with 50 µL of the *L. monocytogenes* strain, previously grown on TSBYE for 24 h at 37 °C. The inoculated semi-solid medium was transferred to the TSB plates, spreading the microorganism over it. After the solidification, 9 paper disks (6 mm diameter) were laid into the plates. Then, 5 µL of the probiotic microorganisms (10^7^ CFU/mL) were discharged on each disk. Distilled water and streptomycin 1 mg/mL were used as negative control (no inhibition) and as a positive control, respectively. Plates were left in ambient conditions approximately for 1 h and subsequently incubated at 37 °C for 22 ± 2 h. The antimicrobial effect was reported by measuring the diameter (mm) of inhibition halos or zones with no microbial growth.

#### 2.2.4. Antimicrobial Activity of Selected Probiotics against *L. monocytogenes* in Juice

The antimicrobial efficacy of probiotic strains against *L. monocytogenes* was tested in PGJ with and without 2% inulin. Probiotic concentrated solutions were produced as described above. Four preselected probiotic microorganisms were assessed in this experiment (LC, LRGG, SYN and BC04). Tested treatments corresponded to PGJ inoculated with *L. monocytogenes* at 10^5^ CFU/mL, PGJ inoculated with the preselected tested probiotic strain at 10^7^ CFU/mL and PGJ inoculated with both *L. monocytogenes* at 10^5^ CFU/mL and the tested probiotic strain (LC, GG, SYN or BC04) at 10^7^ CFU/mL. Inoculated juices were stored in refrigeration conditions (5 ± 1 °C). For each treatment and sampling time, three 12 mL-screw-capped glass tubs were prepared.

Population of *L. monocytogenes* and the probiotic bacteria were determined after inoculation and after 3, 6, 10 and 14 days of storage in triplicate samples. *L. monocytogenes* was evaluated by plating 10-fold dilutions onto PALCAM agar followed by incubation at 37 °C for 24–48 h. The probiotic bacteria were enumerated by plating 10-fold dilutions onto MRS agar or TSA for LAB and BC04, respectively, followed by incubation at 37 °C for 40–48 h. Results were expressed as log CFU/mL.

#### 2.2.5. Antimicrobial Activity of LC Fermentation Products and Postbiotics against *L. monocytogenes* in PGJ with Inulin

PGJ juice + 2% inulin (PGJI) was prepared and divided into two batches. One batch was inoculated with 10^7^ CFU/mL of LC and incubated at 37 °C for 48 h. Afterwards, half of the fermented juice was pasteurized (80 °C, 10 min) in a water bath to kill vegetative cells. A second batch of non-fermented juice was left as a control (no probiotic added). Then, the two batches were inoculated with *L. monocytogenes* to achieve 10^5^ CFU/mL.

The initial concentration of probiotic and *L. monocytogenes* were checked, and the juices were maintained in refrigeration conditions (5 °C). Population of *L. monocytogenes* and the probiotic bacteria were determined after inoculation and after 3, 6, 10 and 14 days of storage in triplicate as indicated above.

#### 2.2.6. Interaction between LC and *S. cerevisiae* and Its Fermentation Metabolites in PGJ with Inulin

The same procedure, as described before, was carried out in order to prepare a PGJI fermented with *L. casei* and PGJI fermented with *L. casei* and subsequently pasteurized. The interaction between *S. cerevisiae* and *L. casei* and its postbiotics (in the pasteurized PGJI) was monitored for 21 days. Beside microbial counts, in the PGJI inoculated with *S. cerevisiae*, ethanol and acetaldehyde were determined as indicators of juice spoilage.

The contents of ethanol and acetaldehyde were determined according to the protocol described by Echeverria et al. [42] At each sampling date, triplicate juice samples (5 mL) were stored at −20 °C until their analysis. Samples were incubated in a water bath at 60 °C and after 60 min, 1 mL samples of the headspace gas were taken with a syringe and injected into an Agilent Technologies 6890 N gas chromatograph (GC) for the determination of both the acetaldehyde and ethanol concentrations. The gas chromatograph was equipped with a flame ionization detector (FID) and a column (2 m × 2 mm i.d.) containing 5% Carbowax on 60/80 Carbopack (Supelco, Bellefonte, PA, USA). The temperature of the injector, detector and oven were 180, 220 and 80 °C, respectively. Concentrations of ethanol and acetaldehyde in the PGJI were calculated using ethanol and acetaldehyde calibration curves prepared by measuring the headspace of Milli-Q water spiked with a known amount of ethanol and acetaldehyde at increasing concentrations and are expressed as mg/L.

### 2.3. Statistical Analysis

Results are expressed by mean ± standard deviation (SD) of 3 repetitions. All data were checked for significant differences by applying analysis of variance test (ANOVA). The criterion for statistical significance was *p* < 0.05. When significant differences were observed, Tukey’s Honest Significant Difference (HSD) of the means was applied. All statistical analysis was carried out using JMP 13 (SAS Institute Inc., Cary, NC, USA).

## 3. Results

### 3.1. Survival of the Probiotics in PG Juice in Refrigeration (5 °C) Conditions and Effect on the Physicochemical Properties

The population of the tested probiotics at 5 °C with and without the prebiotic in peach and grape juice (PGJ) is shown in Table 1. Although every microorganism was inoculated at 10^7^ CFU/mL, only *L. casei* (LC), *L. rhamnosus* GG (LRGG) and *B. coagulans* 04 (BC04) counts remained stable throughout the 21-day period of refrigerated storage. *L. acidophilus* (LA3), *L. reuteri* LR91 (LR91), *L. rhamnosus* SP1 (SP1) and SYNBIO populations decreased significantly throughout storage, regardless of whether or not inulin was present. LC showed the highest viability throughout all of the experiments, and no significant differences in relation to the presence or absence of inulin were observed, from the seventh day of sampling to the end of the experiment. LRGG and BC04 also presented a constant population after 21 days of refrigerated storage, and none of them increased their counts after the addition of inulin.

To be effective, the viability of probiotics in food products is required to be between 10^6^ and 10^7^ CFU/mL or g [43]. As can be seen in the current experiment, clearly, the probiotic viability of formulated probiotic food is linked to the species and type of food matrix [43,44]. Some of the studied lactic acid bacteria strains, SP1, LR91 and LA3, decreased not only significantly but also greatly after 21 days of refrigerated storage. Indeed, these three *Lactobacillus* strains presented a lower count than the above-mentioned criteria for probiotic-enriched food. The stability of *L. acidophilus*, and other LAB strains, has been reported to be affected by different characteristics of the food matrix, such as nutritional composition, water activity, pH, acidity, storage time and conditions (temperature, humidity, oxygen, light …) [45].

In agreement with Min et al. [43], the storage time significantly affected the survivability of *L. acidophilus*. Indeed, among the seven studied microorganisms, LA3 was the most sensitive to the juice conditions, as the population decreased faster, and practically no viable cells were found after 21 days of storage. Although Min et al. [43] found less inhibition in the survival of this microorganism, the fruit matrix might be one of the factors that interfered the most with the survival of this microorganism.

In contrast, SP1, SYNBIO, BC04, LRGG and, to a greater extent, LC reported an optimal stability throughout the storage, with total counts higher than 10^7^ CFU/mL in the final product and at the end of the refrigeration time. LRGG stability in the PGJ is in accordance with other studies that also reported its viability in other fruit matrices, such as pineapple juice [46] and apple [26] stored under refrigerated conditions. *L. casei* was the only microorganism that not only maintained its population throughout storage but also was able to, slightly but significantly, increase it. The suitability of *L. casei* as a probiotic microorganism in fruit matrices has already been studied successfully [47,48].

*B. coagulans* has also been successfully employed in the food industry. Indeed, Kounray et al. [49] were able to develop a probiotic pasta, with a viable probiotic strain after 6 months of storage, even after heat treatment. Even in fruit juices, the implementation of *B. coagulans* has been effective [50].

Although no effect of the addition of inulin in the survival of the probiotics was observed, other authors have reported that the viability of probiotics was enhanced by the addition of inulin to almond milk [51] or oligofructose in clarified apple juice [49].

Physicochemical properties did not change drastically; neither after the addition of probiotic or during the refrigerated storage (Table A1). However, the addition of inulin increased the TSS content in one °Brix. At the beginning of the refrigerated storage period, PGJ without inulin presented the following average physicochemical parameters, independently of the probiotic microorganism used: 3.52, 16.0 °Brix and 4.91 g malic acid/L, for pH, TSS and titratable acidity, respectively. At the beginning of the refrigerated storage period, PGJ without inulin presented the following average physicochemical parameters, independently of the probiotic microorganism used: 3.52, 16.0 °Brix and 4.91 g malic acid/L, for pH, TSS and titratable acidity, respectively. After 21 days of storage at 5 °C, probiotic PGJ barely changed for pH and TSS (3.53 and 16.0 °Brix). However, TA increased progressively with the storage, reaching 5.18 g malic acid/L, also independently of the probiotic. When inulin was added to the PGJ, the average initial values for the physicochemical properties were 3.40, 17.4 °Brix and 4.85 g malic acid/L for the pH, TSS and TA, respectively. After storage, values were similar in the content of TSS and significantly different for the pH and TA (3.53 and 5.17 g malic acid/L, respectively). No effect related to the probiotic was shown. This could be attributed to the fact that the probiotics studied did not grow in the juice under refrigerated conditions. Pimentel et al. [52] evaluated the effect of the supplementation of a clarified apple juice with the probiotic *L. paracasei* with and without oligofructose on the physiochemical characteristics during refrigerated storage (4 °C), describing that the probiotic products presented higher acidity, in comparison with the control juice. Moreover, the addition of oligofructose also increased the TSS content in apple juice.

### 3.2. Survival and Fermentation Capability of the Probiotics in PGJ Juice at 37 °C with or without Inulin and Effect on the Physicochemical Quality

LC and SYNBIO population increased 1-log unit after 72 h of incubation at 37 °C (Table 2). The population of LR91 and LRGG did not show significant differences between day 0 and day 3. However, BC04, SP1 and LA3 population significantly decreased at the end of the fermentation process. No significant differences in population were observed when inulin was added.

Scientific evidence suggests that optimal temperatures would allow *Lactobacillus* bacteria to accelerate their metabolism and thus increase their population. Nevertheless, in this experiment, LR91, LRGG, LA3, SP1 and BC04 did not show any increase in population, but rather decreased after fermentation time. Several factors, such as the food matrix or the strain used, could affect the survival and fermentation capacity of the probiotic. In this regard, opposite to presented results, optimistic results related to the viability and growth of the *L. acidophilus*, in a clarified apple juice [52] and a fermented beetroot and carrot juice [53], were reported. PGJ presents a pH of 4, which is lower than the initial pH of both above-mentioned synbiotic products (over 5) and can explain the non-viability of *L. acidophilus* found in PGJ.

SYNBIO, LRGG and LC were able to maintain their population and even grow under the fermentation conditions proposed. Indeed, LC was the only probiotic that was able to increase their population during fermentation at 37 °C in 1-log unit, after 24 h of fermentation. Likewise, Malik et al. [53], assessed the survival of *L. casei* in a fermented beetroot and in carrot juice, finding an approximately 1-log increase in this microorganism population after 24 h at 35 °C. Similar results, for this microorganism, were also obtained in pineapple juice [54], clarified apple juice [55] and cantaloupe juice [48].

Since *B. coagulans* can be presented in spore form, it is described to be more resistant to high temperatures [56]. Thus, stability in the *B. coagulans* population was expected. Nevertheless, in PGJ, BC04’s population decreased slightly but significantly after 3 days. Opposite results are described in the bibliography, reaching stability, or even growth, after fermentation of cranberry seed fiber with *B. coagulans* [57].

Globally, the viability of none of the tested probiotics was not significantly influenced by the addition of inulin to PGJ. Similarly, in a sea buckthorn and soymilk beverage [58], the addition of inulin at 1 and 3% did not show a significant influence in the growth and viability of *L. casei* ssp. *paracasei*.

In terms of viability results, the *L. casei* population not only remained stable during 21 days of refrigerated storage but also increased under fermentation conditions. Hence, *L. casei* would be the most suitable probiotic for developing a functional PGJ.

Results of physicochemical characterization of the PGJ during incubation at 37 °C are presented in Table 3. As has been seen before, TSS increased from 16.6 ± 0.2 to 17.3 ± 0.3 °Brix due to the addition of inulin and, for some strains, significantly decreased during incubation. Initial pH was in the range from 3.39 to 3.64 and decreased significantly after 72 h, regardless of the presence of inulin in the PGJ fermented with LC, LR91 and LA3, and increased in the case of LR92 and BC04. Initial TA, expressed as g of malic acid/L, ranged from 5.85 to 6.10 and increased after 3 days of incubation to maximum values of 5.85–6.10 in the PGJ-containing LC.

Regarding lactic acid production, D- and L-lactic were determined. However, the main isomer produced was L-lactic (>85%). Thus, results of total lactic acid (D-lactic+L-lactic) are shown (Table 4). *LC* was the probiotic that produced the highest amount of lactic acid, with approximately 3.3 g/L after 24 h and 4.60 to 5.12 after 72 h. LR91 and SYN produced similar amounts of lactic acid (1.8–2.2 g/L). The other strains did not produce significant amounts of lactic acid. Opposite to our findings, significant production of lactic acid was found in date juice after fermentation with *L. rhamnosus* [59]. Although in order to increase the production of lactic acid, typically a source of carbon is used, as reported by the above references, no significant differences were found when the fermentation was carried out with (4.60 ± 0.17 g/L) or without inulin (5.12 ± 0.33 g/L).

Since *BC04* does not belong to the acid-lactic genera, its fermentation did not produce acid lactic, as described by Ohara et al. [60], who reported production of this acid only under anaerobic conditions. However, lactic acid has been reported as the most common metabolite produced by the genera *Bacillus* [5].

According to the results obtained, the production of lactic acid in the juices with *LC* and *SYN* could be explained due to the fermentation process. In addition, LC and SYN were the only probiotics tested in which an increase in the population was observed. pH is one of the physicochemical properties that, along with TA, might be altered after the fermentation process, due to the production of some compounds, mainly acids. In fermented food, lactic acid results from the metabolism of the LAB. Therefore, the decrease in pH reported in the present investigation, at the end of the fermentation process, could be attributed to the production of lactic acid, among other fermentation products. Costa et al. [54] have also reported a reduction in the pH when pineapple juice was fermented with *L. casei.* In the selection of a viable fermentative probiotic in beetroot juice, Baráth et al. [61] also found a drop in pH when the beetroot juice was fermented using lactic acid bacteria.

There is not a specific legislation for fermented juices; however, sanitary specifications establish that fermented milk and acidified milk products must have a titratable acidity of not less than 0.5% expressed as lactic acid and a pH of not more than 4.4 [62]. In this regard, the PGJ fermented with *L. casei*, SP1 and LA3 resulted in values of pH below 4.4 at the end of the fermentation.

Considering the above results, on the one hand, during the refrigerated storage period at 5 °C, SYNBIO, BC04, LRGG and LC survived, without significantly affecting the physicochemical properties of PGJ. On the other hand, if a fermentation process is desired to obtain a different way of delivering the probiotic, LC and SYN could be considered suitable probiotics, as they grew at 37 °C, produced lactic acid and changed the pH and TA of the PGJ.

### 3.3. Antimicrobial Activity of Selected Probiotics against L. monocytogenes

According to the viability, under refrigeration and after fermentation in PGJ, LC, SYN, LRGG and BC04 were selected to evaluate the antimicrobial activity, using two methodologies.

#### 3.3.1. Disk Diffusion Test

Results of the disk diffusion test are shown in Table 5. No in vitro inhibition of any strains of *L. monocytogenes* was observed when testing SYNBIO and BC04. In contrast, LC and LRGG reported the same inhibition rate against each strain of *L. monocytogenes*, ranging from 10 to 15 mm. According to the classification of Abdel-Daim et al. [63], *L. monocytogenes* strains Lm_230/3, CECT-940 and CECT-4032 exhibited a low inhibition by LC and LRGG, whilst a moderate inhibition effect of the strains CECT-933 and CECT-4031 was observed.

Previous studies have attributed the antimicrobial potential of some strains of *Lactobacillus* to several metabolites (e.g., lactic acid, acetic acid, propionic acid) and proteinaceous compounds (bacteriocins) that are able to antagonize pathogens [64,65,66]. Regarding antimicrobial activity, *L. casei* and *L. plantarum* have shown antibacterial activity against *Salmonella* [63,64]. Furthermore, the inhibition effect against *Listeria* of *L. casei* and *L. rhamnosus* was also described by Woo and Ahn [67], agreeing with the results shown by the in vitro test. Likewise, in vivo studies in rats demonstrated that probiotics could enhance host resistance against oral *L. monocytogenes* infection [68]. However, it has been found that probiotics can suppress *L. monocytogenes* colonization to some extent, but never prevent it [69]. Thus, the use of probiotics or their combination, as well as their antibacterial metabolites, has considerable potential in the agri-food industry. Nevertheless, further research should be conducted on the virulence, growth and biofilm formation of pathogens such as *L. monocytogenes* [25].

Contrary to our results, *B. coagulans* had previously been reported to present anti-*Listeria* activity, which has been associated with the production of bacteriocins that alter the cell membrane and DNA of pathogenic bacteria [70]. In food matrices, such as cheese, *B. coagulans* also showed a moderate effect against *L. monocytogenes* [71].

#### 3.3.2. Antimicrobial Effect of LC and LRGG against *L. monocytogenes* in PGJ

**Figure 2:**
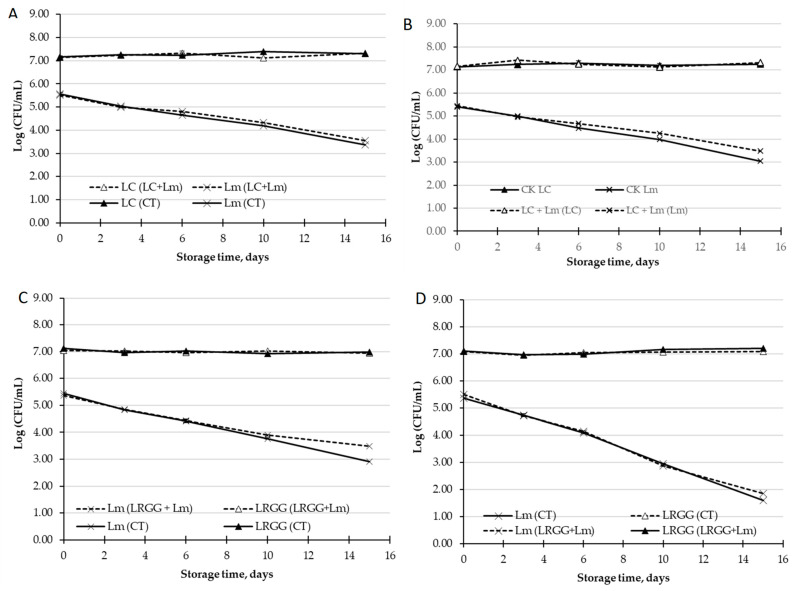
Antimicrobial effect of LC (*L. casei*) and LRGG (*L. rhamnosus* GG) against *L. monocytogenes* (*Lm*) in PGJ. (**A**) Population (log CFU/mL) of LC and Lm in peach and grape juice without inulin. (**B**) Population (log CFU/mL) of LC and Lm in peach and grape juice with inulin. (**C**) Population (log CFU/mL) of LRGG and Lm in peach and grape juice without inulin. (**D**) Population (log CFU/mL) of LRGG and Lm in peach and grape juice with inulin.

Considering the in vitro anti-*Listeria* effect described in the previous section (Section 3.3.1), the anti-*Listeria* effect of LC and LRGG in PGJ was tested. The *L. monocytogenes* population decreased by more than 2-log units throughout the storage in PGJ (Figure 2) in the absence of the probiotics (CT). Neither the presence of LC nor LRGG affected the population of *L. monocytogenes*, and *L. monocytogenes* influenced the viability of these probiotic bacteria. Therefore, it can be suggested that some of the intrinsic characteristics of the PGJ might be responsible for the decrease in this pathogen and not the probiotic’s presence. The low pH and acid content of the PGJ described may justify the antimicrobial effect against *L. monocytogenes* of the control peach and grape juice, without the probiotic strains tested [72]. This behavior has also been reported in other fruit matrices, such as pineapple juice [73]. In addition to the pH, the inhibitory effect of the PGJ on the growth of this pathogen could also be attributed to the high content of some polyphenols with an already-demonstrated antimicrobial effect, such as ferulic acid, resveratrol, gallic and caffeic acid [74].

Opposite to our findings, previous work has shown that the probiotic *L. rhamnosus* GG reduced the population of *L. monocytogenes* in cut apples, without affecting the product’s quality [26,27]. Russo et al. [26,27] also demonstrated that two potentially probiotic strains (*Lactobacillus plantarum* B2 and *Lactobacillus fermentum* PBCC11.5) decreased the population of *L. monocytogenes* in pineapple and melon. Yang et al. [75] also described the effectiveness of LAB against inoculated *L. innocua* in cut onions.

On the one hand, *LC* presented the highest survival rate, even growth, both after refrigerated storage (<0.5-log after 21 days) and after fermentation (>1-log after 3 days); it produced lactic acid to a greater extent (4.64 g/L after 3 days of fermentation) and, additionally, exhibited an in vitro antimicrobial effect against *L. monocytogenes*, and it was the selected probiotic for the formulation of the synbiotic PGJ. Furthermore, considering that population and lactic acid production did not show significant differences between the 2nd and 3rd day of fermentation, a 2-day fermentation time was preferred for subsequent analysis. On the other hand, the addition of inulin, as a prebiotic, did not result in any positive effect on the above determinations. However, it was the already-referenced positive impact of inulin on the microbiota and, consequently, on the health status of consumers which suggested the formulation of the synbiotic PGJ with inulin.

#### 3.3.3. Evaluation of the Antimicrobial Activity of the *L. casei* Fermentation Products and Postbiotics against *L. monocytogenes* in PGJI

**Figure 3:**
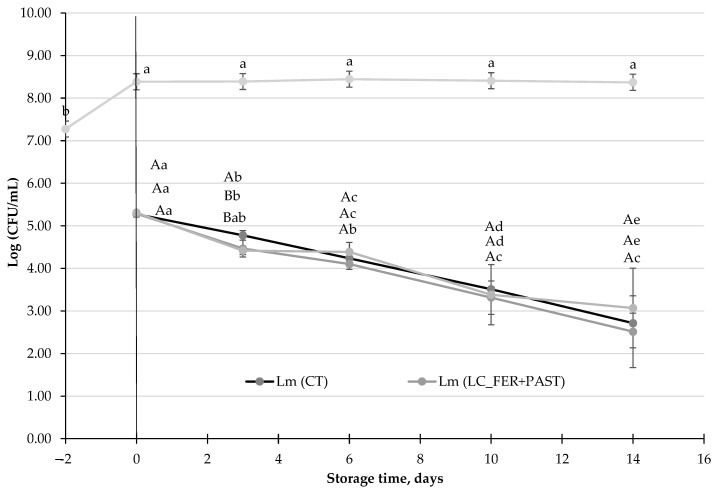
*L. monocytogenes* (Lm) population (CFU/mL) in a *L. casei*-fermented Lm (LC_FER), *L. casei*-fermented and pasteurized Lm (LC_FER+PAST) peach and grape juice with 2% inulin (PGJI), and in a PGJI without the probiotic, Lm (CT). LC (LC_FER) shows the population of LC (log CFU/mL) in fermented and non-pasteurized PGJI throughout 21 days of storage at 5 °C. For each day of storage, different capital letters indicate significant differences among distinct treatments. For each juice, different lowercase letters indicate significant differences between the different days of storage according to an ANOVA test (*p* < 0.05).

This experiment aimed to determine whether the metabolites produced during the fermentation of PJG with 2% inulin (PGJI), either pasteurized or unpasteurized, have antimicrobial activity. As previously described, *L. monocytogenes* population decreased 2.5-log units in PGJI, after 14 days of storage under refrigeration conditions, and the presence of *L. monocytogenes* did not affect the survival of *LC*. Moreover, neither the probiotic itself nor the metabolites produced during the fermentation enhanced the decrease in the pathogen (Figure 3). Postbiotic release, in PGJI after pasteurization, exhibited no antimicrobial effect either.

While as described above, some probiotics may exhibit an inhibitory effect against some microorganisms, it has also been suggested that postbiotic compounds might enhance the antimicrobial functionality of probiotics [76]. A postbiotic can be defined as a compound that usually belongs to the structure of a probiotic microorganism and is released after its degradation, manifesting, consequently, its beneficial effect when the microorganism is not alive [27]. Although in this research, no postbiotic effect has been described in pasteurized PGJ, after fermentation with *L. casei*, some postbiotics with antimicrobial effect from LAB have already been identified [75].

### 3.4. Effect of LC and LC Postbiotics against S. cerevisiae Population, Ethanol and Acetaldehyde Production in PGJI

**Figure 4:**
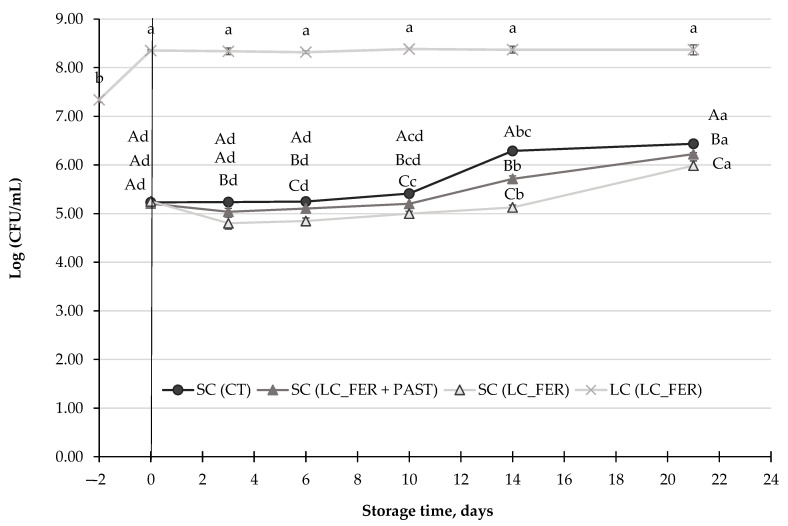
*S. cerevisiae* population (SC) (log CFU/mL) in a *L. casei*-fermented SC (LC_FER), *L. casei*-fermented and pasteurized SC (LC_FER+PAST) PGJI and in PGJI without LC throughout 21 days of storage at 5 °C. *L. casei* (LC) population (CFU/mL) in fermented and non-pasteurized PGJI LC (LC_FER) is also shown. For each day of storage, different capital letters indicate significant differences among distinct treatments. For each juice, different lowercase letters indicate significant differences between the different days of storage according to an ANOVA test (*p* < 0.05).

*S. cerevisiae* is one of the yeasts typically associated with the spoilage of fruit juices [77]. Results demonstrated that the yeast was able to grow in the PGJI during storage at 5 °C (Figure 4), increasing its population in 1-log unit, after 21 days. A slight, but significant, inhibition (lower than 0.5 log units) was observed in the *S. cerevisiae* population in the fermented PGJI, regardless of whether the juice had been pasteurized or not. Similar results were obtained in grape beverages [78].

As mentioned before, some bioactive compounds, naturally present in fruits and their derived products, such as polyphenols, have been reported to exhibit antimicrobial activity. However, the behavior of polyphenols against microbes depends on the group and structure of the bioactive compound, as well as on the species and genera to which the microorganism belongs [79]. Due to the composition of their cell wall, yeasts are more resistant to the antimicrobial activity of polyphenols than other microorganisms. In addition, carbon-rich compounds in juices, such as sugars [80,81], can be a source of nutrients for *S. cerevisiae*, which could contribute to its growth from day 10 onwards. Indeed, Cheirsilp et al. [82] mentioned the possibility that *S. cerevisiae* can consume lactic acid produced by *Lactobacillus* under aerobic conditions. However, there was no greater inhibition in the combination of *S. cerevisiae* with the probiotic or vice versa. In this regard, the effect of the presence of *S. cerevisiae* on the viability of a *Lactobacillus* strain has been tested in sourdough [83], with no negative effects on the lactic acid bacteria community, according to the results of this experiment.

Despite the potentially antimicrobial effect of probiotics against pathogenic bacteria, there are not enough studies referring to the inhibitory effect of probiotics against yeasts and molds. Indeed, the treatment with LAB on onions had no significant effect against these microorganisms [75]. The slight but significant effect of fermented juice against yeast, observed in this study, would not allow for considering the treatment with *L. casei* as an effective management tool of *S. cerevisiae*. For an agent or treatment to be considered effective against microorganisms in the food industry, a minimum reduction of two logarithmic cycles is required [84].

**Figure 5:**
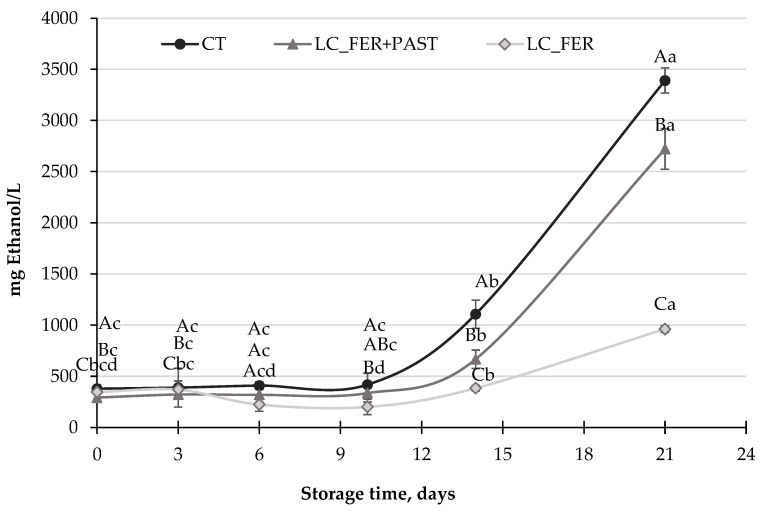
Ethanol production (mg/L) by *S. cerevisiae* in a PGJI fermented with *L. casei* (LC_FER), PGJI fermented with LC and pasteurized (LC_FER+PAST) and in a PGJI without the probiotic (CT) throughout 21 days of storage at 5 °C. For each day of storage, different capital letters indicate significant differences among distinct treatments. For each juice, different lowercase letters indicate significant differences between the different days of storage according to an ANOVA test (*p* < 0.05).

Ethanol production by *S. cerevisiae* in PGJI increased significantly after 14 days of storage at 5 °C (Figure 5), reaching levels of up to almost 3500 mg ethanol/L (0.35%) in the last six days of refrigerated storage. Budak et al. [62] reported higher ethanol production (7.6%) after fermentation of a peach beverage by *S. cerevisiae* at 30 °C, supporting the potential of the peach matrix to be fermented by *S. cerevisiae*. *LC*-fermented PGJI contained lower amounts of ethanol at the end of storage, with less than 2500 mg/L (0.25%) for fermented and pasteurized PGJI and 1000 mg/L (0.1%) for fermented PGJI. Therefore, and despite the minimal impact of LC fermentation against the population of *S. cerevisiae* in PGJI, a positive effect in reducing the production of one of the most unpleasant substances (ethanol) produced by *S. cerevisiae* could be pointed out. Indeed, the inhibitory effect of *Lactobacillus* and *Bifidobacterium,* and in particular of *L. casei*, on ethanol production by *S. cerevisiae* has already been reported by Bassi et al. [85], in broths fermented at the end of the sixth fermentation cycle. Agreeing with these results, an inhibition of ethanol delivery by *S. cerevisiae* has also been reported in probiotic sourdough [83]. However, not all *Lactobacillus* strains have been described as inhibitors of ethanol bioproduction, as described by Carvalho et al. [86].

**Figure 6:**
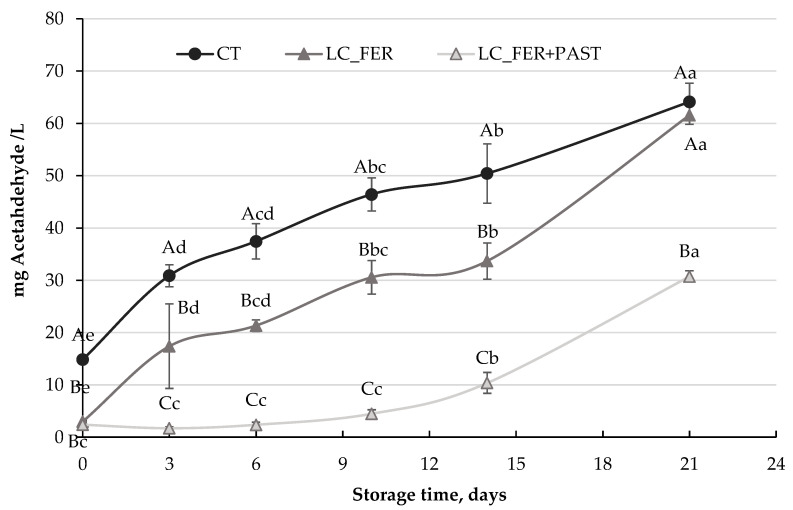
Acetaldehyde production (mg/L) by *S. cerevisiae* contamination in a *L. casei*-fermented (LC_FER), *L. casei*-fermented and pasteurized (LC_FER+PAST) PGJ with inulin (PGJI) and in the PGJI without LC (CT) throughout 21 days of storage at 5 °C. For each day of storage, different capital letters indicate significant differences among distinct treatments. For each juice, different lowercase letters indicate significant differences between the different days of storage according to an ANOVA test (*p* < 0.05).

Although fermentation of PGJI with *Lactobacillus casei* did not achieve substantial inhibition of *S. cerevisiae*, Figure 6 shows the reduction in acetaldehyde production in PGJI fermented with *L. casei* and contaminated with *S. cerevisiae*. While *S. cerevisiae*, in the absence of the probiotic, produced more than 60 mg acetaldehyde/L after 21 days, the fermentation process reduced the production of this unpleasant metabolite by half (30 mg/L) at the end of the storage period. PGJI fermented with *L casei* and pasteurized also presented lower values of this metabolite during storage, but at the end of the refrigeration time, similar values to the control treatment were obtained. Hence, the reduction in the acetaldehyde yield would appear to be more associated with the presence of viable LC cells.

Acetaldehyde is a product of the metabolism of several microorganisms that contributes to aroma, color, and microbiological stability and may be attributed to dairy products [87], but it is also involved in alcoholic fermentation carried out by *S. cerevisiae*. It can appear in foods and beverages other than wine, such as juices, and can be an indicator of the presence of spoilage microorganisms, such as *S. cerevisiae*. While this aroma compound can be desirable in some fermented products, acetaldehyde grassy or oxidized aroma can be considered unpleasant [88]. Furthermore, the presence of this compound has been related to some carcinogenic processes [89]. The function of acetaldehyde is to serve as an electron acceptor, which can be used in later stages of fermentation by its producer, mainly *S. cerevisiae* [88]. Considering the unpleasant aroma attributed to this metabolite, its unhealthy properties, and the fact that it can be used as a substrate by microorganisms (*S. cerevisiae*), it would be advisable to minimize its production.

Acetaldehyde content in the PGJI fermented and pasteurized was higher than that observed in unpasteurized PGJI. This may be explained not only because of the potentially lower pH of the fermented juice containing viable probiotic cells but also because the probiotic may act like a direct competitor of the substrate (glucose) that *S. cerevisiae* utilizes to produce acetaldehyde [90].

Regarding the results presented, the antimicrobial potential of *L. casei* did not result in the inhibition of the growth of the pathogen or the spoilage microorganism but in the reduction in metabolite production, after contamination with the spoilage microorganism, under the conditions and in the food matrix studied.

## 4. Conclusions

Recently, probiotics have gained significant attention from consumers and the agrifood industry. The lately reported health promotion properties are boosting pro- and prebiotics in the functional food market. Although there are currently many products rich in probiotics, dairy products, including fermented dairy products, are widely used as probiotic carriers. However, current dietary trends involving health and environmental concerns are encouraging the food industry to research and design plant-based products that are capable of delivering probiotics in a safe and healthy matrix. In this regard, it is necessary to identify probiotics, strategies to ensure viability, study their functionality and design products that can effectively host and deliver probiotics.

Likewise, this research focused on the study of the viability of seven probiotic cultures in a non-dairy matrix, peach and grape juice (PGJ), under refrigerated and fermented conditions. Furthermore, an extensive evaluation of the antimicrobial activity of the selected probiotic against *L. monocytogenes* and *S. cerevisiae*, as well as against their metabolites, was carried out in synbiotic PGJ.

Out of the seven probiotics screened in PGJ, *L. casei*, *L. rhamnosus* GG, SYNBIO and *B. coagulans* 04 showed the highest viability under refrigerated storage and after fermentation. Additionally, *L casei* and *L. rhamnosus* GG showed anti-*Listeria* activity under in vitro conditions. Due to its viability under both studied conditions, lactic acid production and anti-*Listeria* activity, *L. casei* was selected to develop a synbiotic PGJ. Although fermentation or postbiotic release after pasteurization did not produce antimicrobial inhibition, ethanol and acetaldehyde production by *S. cerevisiae* was reduced after fermentation of PGJ with *L. casei* for 48 h, in the presence of inulin.

Consequently, and due to its viability and probiotic potential in PGJ, *L. casei* could be proposed as a suitable probiotic culture to develop a synbiotic PGJ. Further research is required to determine the effect of *L. casei* on the quality and functionality of the synbiotic PGJ, as well as to determine its shelf life.

## Figures and Tables

**Table 1:** Probiotic viability (log CFU/mL) in the PG juices with or without (Ø) inulin throughout refrigerated storage time.

		Time of Storage (5 °C), Days				
Probiotic	Prebiotic	0	3	7	15	21
LC	Inulin	7.19 ± 0.07 c	7.28 ± 0.08 bc	7.43 ± 0.04 ab	7.37 ± 0.19 abc	7.54 ± 0.02 a
	Ø Inulin	7.29 ± 0.07 a	7.14 ± 0.03 a	7.32 ± 0.09 a	7.33 ± 0.02 a	7.60 ± 0.01 a
SP1	Inulin	6.40 ± 0.06 a	5.11 ± 0.04 b	2.51 ± 0.09 c	1.92 ± 0.03 d	1.47 ± 0.07 e *
	Ø Inulin	6.41 ± 0.04 a	5.14 ± 0.09 b	2.51 ± 0.06 c	2.00 ± 0.13 d	1.84 ± 0.09 d
SYN	Inulin	6.31 ± 0.25 a	6.11 ± 0.06 ab	5.95 ± 0.08 b	5.56 ± 0.07 c	5.45 ± 0.05 c
	Ø Inulin	6.22 ± 0. 11 a	6.19 ± 0.01 a	6.03 ± 0.01 b	5.64 ± 0.02 c	5.49 ± 0.05 d
LR91	Inulin	6.83 ± 0.07 a *	6.25 ± 0.06 b *	5.96 ± 0.03 b	4.58 ± 0.23 c *	3.21 ± 0.07 d *
	Ø Inulin	6.47 ± 0.06 a	6.37 ± 0.05 a	6.35 ± 0. 52 a	5.55 ± 0.03 b	5.02 ± 0.28 b
LA3	Inulin	6.37 ± 0.00 a *	5.42 ± 0.16 b *	5.22 ± 0.29 b *	3.39 ± 0.28 c *	0.40 ± 0.00 d *
	Ø Inulin	6.34 ± 0.01 a	6.21 ± 0.05 a	5.95 ± 0. 32 a	3.99 ± 0.13 b	0.55 ± 0.21 c
LRGG	Inulin	7.30 ± 0.06 a	7.25 ± 0. 11 a *	7.22 ± 0.03 a	7.22 ± 0.08 a	7.15 ± 0.02 b
	Ø Inulin	7.23 ± 0.08 a	7.09 ± 0. 19 a	7.26 ± 0.061 a	7.42 ± 0. 13 a	7.17 ± 0.04 a
BC04	Inulin	6.30 ± 0. 25 a	6.13 ± 0. 12 a	6.15 ± 0.08 a	6.54 ± 0.05 a	6.25 ± 0. 22 a
	Ø Inulin	6.48 ± 0.03 a	6.04 ± 0.21 b	6.05 ± 0.04 b	6.75 ± 0. 12 a	6.59 ± 0.2 a

Values correspond to the mean of 3 replicates ± standard deviation. For each probiotic and storage time, * denotes significant differences between treatments (with and without inulin) and different lowercase letters denote significant differences among days (*p* < 0.05).

**Table 2:** Probiotic viability (log CFU/mL) in the PG juices with or without (Ø) inulin throughout fermentation time.

		Time of Fermentation (37 °C), Days			
Probiotic	Prebiotic	0	1	2	3
LC	Inulin	7.19 ± 0.07 b	8.17 ± 0.05 a	8.22 ± 0.09 a	8.38 ± 0.04 a *
	Ø Inulin	7.29 ± 0.07 c	8.12 ± 0.13 b	8.10 ± 0.17 ab	8.26 ± 0.02 a *
SP1	Inulin	6.40 ± 0.06 a	3.09 ± 0.00 b	3.09 ± 0.00 b	3.79 ± 0.73 b
	Ø Inulin	6.41 ± 0.04 a	3.09 ± 0.00 b	3.09 ± 0.00 b	3.61 ± 1.09 b
SYN	Inulin	6.31 ± 0.25 b	5.41 ± 0.05 c	6.22 ± 0.03 b	7.15 ± 0.09 a
	Ø Inulin	6.22 ± 0.11 b	5.48 ± 0.02 c	6.19 ± 0.13 b	7.15 ± 0.05 a
LR91	Inulin	6.83 ± 0.07 a *	7.02 ± 0.09 a	6.39 ± 0.10 b	6.11 ± 0.29 b
	Ø Inulin	6.47 ± 0.06 b	6.91 ± 0.04 a	6.41 ± 0.04 b	5.79 ± 0.11 c
LA3	Inulin	6.37 ± 0.00 a *	3.05 ± 0.00 b	2.70 ± 0.61 b	0.70 ± 0.03 c *
	Ø Inulin	6.34 ± 0.01 a	3.05 ± 0.00 b	2.32 ± 0.97 b	1.65 ± 0.90 b
LRGG	Inulin	7.30 ± 0.06 ab	7.40 ± 0.04 ab *	7.56 ± 0. 16 a	7.29 ± 0.09 b *
	Ø Inulin	7.23 ± 0.08 b	7.58 ± 0. 10 a	7.37 ± 0.16 c	7.56 ± 0.01 a
BC04	Inulin	6.30 ± 0. 25 a	5.61 ± 0.10 bc	5.85 ± 0.10 ab	6.02 ± 0.03 c *
	Ø Inulin	6.48 ± 0.03 a	5.77 ± 0.17 b	5.78 ± 0.00 b	5.55 ± 0.09 b

Values correspond to the mean of 3 replicates ± standard deviation. For each probiotic and storage time, an * denotes significant differences between treatments (with and without inulin) and different lowercase letters denote significant differences between days (*p* < 0.05).

**Table 3:** Physicochemical parameters of the PGJ with or without (Ø) inulin, inoculated with the probiotic cultures and incubated at 37 °C.

		Time of Fermentation (37 °C), Days											
		pH				SST				TA			
Probiotic	Prebiotic	0	1	2	3	0	1	2	3	0	1	2	3
LC	Inulin	3.44 ± 0.01 b	3.40 ± 0.01 c	3.55 ± 0.00 a	3.20 ± 0.03 d	17.20 ± 0.00 a	17.23 ± 0.06 a	17.27 ± 0.06 a	16.70 ± 0.1 b	5.03 ± 0.08 b	4.36 ± 0.11 c	5.03 ± 0.06 b	5.85 ± 0.15 a
	Ø Inulin	3.43 ± 0.02 b	3.42 ± 0.02 b	3.47 ± 0.02 a	3.16 ± 0.01 c	15.70 ± 0.00 b	15.83 ± 0.06 a	15.64 ± 0.06 b	15.10 ± 0.00 c	5.14 ± 0.15 b	4.64 ± 0.09 c	5.15 ± 0.06 b	6.10 ± 0.09 a
SP1	Inulin	3.43 ± 0.03 b	3.42 ± 0.02 b	3.55 ± 0.01 a	3.24 ± 0.02 c	17.33 ± 0.06 b	17.40 ± 0.00 ab	17.57 ± 0.12 a	16.77 ± 0.06 c	5.17 ± 0.13 a	4.78 ± 0.05 b	4.77 ± 0.06 b	4.89 ± 0.12 ab
	Ø Inulin	3.54 ± 0.01 a	3.46 ± 0.02 b	3.51 ± 0.01 a	3.25 ± 0.01 c	16.13 ± 0.06 a	15.93 ± 0.06 b	16.13 ± 0.06 a	15.370 ± 0.12 c	5.06 ± 0.23 a	4.88 ± 0.05 a	4.82 ± 0.10 a	4.86 ± 0.23 a
SYN	Inulin	3.61 ± 0.01 ab	3.58 ± 0.03 b	3.52 ± 0.01 c	3.46 ± 0.00 d	17.57 ± 0.06 a	17.53 ± 0.12 a	13.30 ± 0.00 b	17.43 ± 0.06 ab	4.75 ± 0.1 a	4.77 ± 0.06 a	4.34 ± 0.25 b	4.32 ± 0.12 b
	Ø Inulin	3.64 ± 0.02 a	3.50 ± 0.07 b	3.60 ± 0.03 a	3.62 ± 0.01 a	16.13 ± 0.06 a	15.97 ± 0.12 ab	15.80 ± 0.00 bc	15.77 ± 0.06 c	4.77 ± 0.02 a	4.75 ± 0.05 a	4.32 ± 0.05 b	4.28 ± 0.13 b
LR92	Inulin	3.39 ± 0.01 c	3.50 ± 0.01 b	3.65 ± 0.02 a	3.62 ± 0.03 a	17.83 ± 0.06 a	17.23 ± 0.06 c	17.50 ± 0.1 b	17.53 ± 0.12 b	4.73 ± 0.02 b	5.43 ± 0.17 a	5.42 ± 0.12 a	5.36 ± 0.09 a
	Ø Inulin	3.39 ± 0.01 c	3.52 ± 0.02 b	3.68 ± 0.02 a	3.54 ± 0.02 b	16.20 ± 0.00 a	15.73 ± 0.06 b	16.13 ± 0.06 a	15.73 ± 0.06 b	4.80 ± 0.01 b	5.43 ± 0.11 a	5.44 ± 0.14 a	5.27 ± 0.10 a
LA-3	Inulin	3.51 ± 0.01 a	3.50 ± 0.02 ab	3.47 ± 0.01 b	3.39 ± 0.02 c	17.10 ± 0.00 d	17.20 ± 0.00 c	17.57 ± 0.06 a	17.37 ± 0.06 b	4.52 ± 0.03 a	4.74 ± 0.06 a	4.64 ± 0.09 a	4.65 ± 0.24 a
	Ø Inulin	3.58 ± 0.01 a	3.50 ± 0.01 b	3.54 ± 0.03 a	3.43 ± 0.01 c	15.93 ± 0.10 a	15.90 ± 0.00 a	15.90 ± 0.06 a	15.80 ± 0.00 a	4.73 ± 0.08 a	4.588 ± 0.07 a	4.88 ± 0.14 a	4.82 ± 0.11 a
LRGG	Inulin	3.59 ± 0.02 b	3.66 ± 0.01 a	3.61 ± 0.04 ab	3.51 ± 0.00 c	17.40 ± 0.10 a	16.37 ± 0.12 a	17.13 ± 0.06 a	16.33 ± 0.06 a	4.84 ± 0.13 a	4.30 ± 0.09 c	4.85 ± 0.16 a	4.61 ± 0.06 b
	Ø Inulin	3.58 ± 0.01 b	3.68 ± 0.02 a	3.55 ± 0.01 bc	3.54 ± 0.02 c	15.90 ± 0.00 a	15.43 ± 0.06 b	15.20 ± 0.00 c	15.33 ± 0.06 b	4.83 ± 0.17 a	4.26 ± 0.09 c	4.94 ± 0.26 a	4.46 ± 0.08 b
BC04	Inulin	3.43 ± 0.01 c	3.73 ± 0.05 b	3.77 ± 0.01 a	3.79 ± 0.02 a	17.70 ± 0.00 a	17.73 ± 0.06 a	17.80 ± 0.10 a	17.70 ± 0.00 a	4.94 ± 0.06 a	4.69 ± 0.21 a	4.97 ± 0.04 a	4.91 ± 0.06 a
	Ø Inulin	3.46 ± 0.01 c	3.72 ± 0.02 b	3.72 ± 0.01 b	3.80 ± 0.03 a	16.33 ± 0.06 ab	16.40 ± 0.00 a	16.17 ± 0.06 c	16.23 ± 0.06 bc	5.02 ± 0.04 ab	4.74 ± 0.24 b	5.14 ± 0.07 a	4.96 ± 0.03 ab

Values of total soluble solids (TSS, °Brix), titratable acidity (TA, g malic acid/L of juice) and pH correspond to the mean of 3 replicates ± standard deviation. For the same parameter and each probiotic, different lowercase letters denote significant differences between days (*p* < 0.05).

**Table 4:** Lactic acid concentration (g total lactic acid/L) in PGJ with or without (Ø) inulin inoculated with the probiotic strains and incubated at 37 °C.

		Time at 37 °C, Days			
Probiotic	Prebiotic	0	1	2	3
LC	Inulin	0.11 ± 0.01 c	3.31 ± 0.10 b	4.34 ± 0. 15 a	4.60 ± 0. 17 a
	Ø Inulin	0.10 ± 0.01 d	3.31 ± 0.09 c	4.19 ± 0.06 b	5.12 ± 0. 33 a
SP1	Inulin	0.11 ± 0.02 a	0.11 ± 0.01 a	0.12 ± 0.01 a	0.11 ± 0.01 a
	Ø Inulin	0.11 ± 0.01 a	0.10 ± 0.01 a	0.13 ± 0.02 a	0.10 ± 0.01 a
SYNBIO^®^	Inulin	0.12 ± 0.01 d	0.18 ± 0.01 c	0.83 ± 0.13 b	1.78 ± 0.02 a
	Ø Inulin	0.11 ± 0.00 d	0.19 ± 0.01 c	1.17 ± 0.09 b *	1.82 ± 0.05 a
LR91	Inulin	0.12 ± 0.01 c	0.99 ± 0.05 b	1.88 ± 0.07 a	1.72 ± 0. 23 a
	Ø Inulin	0.11 ± 0.00 c	1.02 ± 0.17 b	1.98 ± 0. 14 a	2.20 ± 0.09 a
LA3	Inulin	0.12 ± 0.01 a	0.07 ± 0.02 b	0.07 ± 0.01 b	0.07 ± 0.02 b
	Ø Inulin	0.11 ± 0.00 a	0.07 ± 0.01 b	0.06 ± 0.01 b	0.06 ± 0.01 b
LRGG	Inulin	0.03 ± 0.01 a	0.08 ± 0.00 a	0.10 ± 0.05 a	0.22 ± 0.05 a
	Ø Inulin	0.04 ± 0.01 a	0.09 ± 0.01 a	0.29 ± 0.07 a *	0.39 ± 0.01 a *
BC04	Inulin	0.05 ± 0.01 b	0.07 ± 0.01 a	0.07 ± 0.00 ab	0.08 ± 0.00 b
	Ø Inulin	0.05 ± 0.01 b	0.05 ± 0.01 ab	0.06 ± 0.00 ab	0.07 ± 0.01 b

Values correspond to the mean of 3 replicates ± standard deviation. For each probiotic and storage day, an * denotes significant differences between treatments (with and without inulin). For each treatment, different lowercase letters denote significant differences between days (*p* < 0.05).

**Table 5:** In vitro antimicrobial activity of four selected probiotics (LC, LRGG, SYNBIO and BC04) against five strains of *L. monocytogenes*.

*L. monocytogenes* Strain	Streptomycin	Water	LC	LRGG	SYNBIO	BC04
Lm_230/3	12.0	0	10.0	10.0	0	0
Lm_933	16. 7	0	11.3	11.3	0	0
Lm_940	14.0	0	10.0	10.0	0	0
Lm_4031	17.7	0	12.0	12.0	0	0
Lm_4032	12.3	0	10.0	10.0	0	0

Values are expressed in mm of inhibition halo and correspond to the mean of 3 replicates.

## Data Availability

The original contributions presented in the study are included in the article, further inquiries can be directed to the corresponding author.

## Appendix A

**Table A1:** Physicochemical parameters of the PGJ inoculated with the probiotic cultures and incubated at 5 °C. Values of total soluble solids (TSS, °Brix), titratable acidity (TA, g malic acid/L of juice) and pH correspond to the mean of 3 replicates ± standard deviation. For the same parameter and each probiotic, different lowercase letters denote significant differences between days (*p* < 0.05).

		Time of Fermentation (37 °C), Days														
		pH					SST					TA				
Probiotic	Prebiotic	0	3	7	14	21	0	3	7	14	21	0	3	7	14	21
LC	Inulin	3.44 ± 0.01 bc	3.37 ± 0.02 c	3.61 ± 0.04 a	3.44 ± 0.01 bc	3.47 ± 0.05 b	17.20 ± 0.00 a	16.23 ± 0.06 a	17.17 ± 0.06 a	17.23 ± 0.06 a	17.23 ± 0.06 a	5.03 ± 0.08 a	4.75 ± 0.10 c	4.67 ± 0.09 c	4.80 ± 0.04 bc	4.96 ± 0.14 ab
	Ø Inulin	3.43 ± 0.02 b	3.42 ± 0.04 bc	3.53 ± 0.02 a	3.36 ± 0.04 c	3.51 ± 0.01 a	15.70 ± 0.00 bc	15.57 ± 0.06 c	15.80 ± 0.01 ab	15.97 ± 0.12 a	15.90 ± 0.00 a	5.14 ± 0.15 a	4.69 ± 0.08 b	4.900.14 ab	4.920 ± 0.05 ab	4.90 ± 0.07 ab
SP1	Inulin	3.43 ± 0.03 bc	3.42 ± 0.03 c	3.56 ± 0.02 a	3.50 ± 0.02 abc	3.50 ± 0.04 ab	17.33 ± 0.06 c	17.20 ± 0.00 d	17.50 ± 0.00 b	17.33 ± 0.06 c	17.67 ± 0.06 a	5.17 ± 0.13 a	4.71 ± 0.08 b	4.76 ± 0.06 ab	4.76 ± 0.27 ab	4.81 ± 0.03 ab
	Ø Inulin	3.54 ± 0.01 a	3.34 ± 0.02 c	3.48 ± 0.01 b	3.46 ± 0.02 a	3.53 ± 0.01 a	16.13 ± 0.06 a	15.60 ± 0.00 b	16.10 ± 0.00 a	15.63 ± 0.06 b	16.03 ± 0.06 a	5.060.23 a	4.92 ± 0.16 a	4.86 ± 0.25 a	4.87 ± 0.14 a	4.86 ± 0.11 a
SYN	Inulin	3.62 ± 0.01 a	3.58 ± 0.00 b	3.52 ± 0.01 c	3.49 ± 0.00 d	3.46 ± 0.00 e	17.57 ± 0.06 a	17.47 ± 0.06 a	17.43 ± 0.06 a	17.47 ± 0.06 a	17.27 ± 0.06 b	4.75 ± 0.1 ab	4.550.07 b	4.55 ± 0.10 b	4.55 ± 0.07 b	4.810.10 a
	Ø Inulin	3.64 ± 0.02 a	3.57 ± 0.01 b	3.54 ± 0.01 bc	3.52 ± 0.02 c	3.48 ± 0.01 d	16.13 ± 0.06 a	15.83 ± 0.06 c	15.97 ± 0.06 bc	16.00 ± 000 ab	15.87 ± 0.06 bc	4.77 ± 0.02 ab	4.68 ± 0.06 b	4.73 ± 0.06 ab	4.72 ± 0.02 b	4.89 ± 0.10 a
LR92	Inulin	3.39 ± 0.01 c	3.52 ± 0.01 a	3.54 ± 0.01 a	3.43 ± 0.01 b	3.44 ± 0.01 b	17.83 ± 0.06 a	17.33 ± 0.06 bc	17.50 ± 0.01 b	17.27 ± 0.06 c	17.27 ± 0.06 c	4.73 ± 0.02 b	4.82 ± 0.01 b	4.78 ± 0.09 b	4.86 ± 0.1 b	7.14 ± 0.08 a
	Ø Inulin	3.39 ± 0.01 c	3.47 ± 0.01 b	3.52 ± 0.01 a	3.46 ± 0.01 b	3.39 ± 0.01 c	16.20 ± 0.00 a	15.70 ± 0.00 c	16.03 ± 0.03 b	16.00 ± 0.00 b	15.73 ± 0.06 c	4.80 ± 0.01 b	4.84 ± 0.04 b	4.91 ± 0.16 b	4.79 ± 0.19 b	7.20 ± 0.29 a
LA-3	Inulin	3.51 ± 0.01 a	3.50 ± 0.02 a	3.50 ± 0.03 a	3.50 ± 0.04 a	3.43 ± 0.00 b	17.10 ± 0.00 c	17.20 ± 0.00 b	17.50 ± 0.00 a	17.23 ± 0.06 b	17.23 ± 0.06 b	4.52 ± 0.03 a	4.75 ± 0.02 a	4.60 ± 0.09 a	4.52 ± 0.20 a	4.70 ± 0.16 a
	Ø Inulin	3.58 ± 0.01 a	3.51 ± 0.01 a	3.48 ± 0.02 a	3.48 ± 0.01 a	3.48 ± 0.01 a	15.90 ± 0.1 b	15.56 ± 0.06 c	16.17 ± 0.06 a	15.80 ± 0.00 b	15.90 ± 0.00 b	4.73 ± 0.08 ab	4.74 ± 0.12 ab	4.82 ± 0.02 a	4.58 ± 0.08 b	4.66 ± 0.03 ab
LRGG	Inulin	3.59 ± 0.02 bc	3.57 ± 0.01 c	3.60 ± 0.01 bc	3.61 ± 0.01 b	3.65 ± 0.01 a	17.40 ± 0.10 a	17.07 ± 0.06 a	17.10 ± 0.00 a	17.17 ± 0.06 a	17.50 ± 0.16 a	4.84 ± 0.13 b	5.08 ± 0.06 a	5.06 ± 0.06 a	5.12 ± 0.04 a	5.02 ± 0.05 a
	Ø Inulin	3.58 ± 0.01 c	3.63 ± 0.01 ab	3.60 ± 0.01 bc	3.65 ± 0.01 a	3.61 ± 0.02 a	15.90 ± 0.00 a	15.630.0 b	15.53 ± 0.06 b	15.83 ± 0.06 a	15.67 ± 0.06 b	5.17 ± 0.02 a	4.83 ± 0.17 b	5.06 ± 0.15 ab	5.18 ± 0.23 ab	5.10 ± 0.03 ab
BC04	Inulin	3.43 ± 0.01 c	3.73 ± 0.03 bb	3.76 ± 0.03 a	3.65 ± 0.02 b	3.74 ± 0.01 b	17.70 ± 0.00 a	17.700.01 a	17.70 ± 0.00 a	17.70 ± 0.00 a	17.87 ± 0.06 a	4.94 ± 0.06 ab	4.83 ± 0.08 bc	5.06 ± 0.02 a	4.68 ± 0.04 c	4.72 ± 0.08 c
	Ø Inulin	3.46 ± 0.01 c	3.73 ± 0.03 bb	3.80 ± 0.03 a	3.70 ± 0.0 b2	3.74 ± 0.01 b	16.33 ± 0.06 b	16.33 ± 0.06 b	16.67 ± 0.06 a	16.67 ± 0.06 a	16.63 ± 0.06 a	5.02 ± 0.04 ab	5.05 ± 0.04 a	5.06 ± 0.07 a	4.92 ± 0.22 ab	4.68 ± 0.10 b

## References

[1] Naseem Z., Mir S.A., Wani S.M., Rouf M.A., Bashir I., Zehra A. (2023). Probiotic-fortified fruit juices: Health benefits, challenges, and future perspective. Nutrition.

[2] Jankovic I., Sybesma W., Phothirath P., Ananta E., Mercenier A. (2010). Application of probiotics in food products-challenges and new approaches. Curr. Opin. Biotechnol..

[3] Hill C., Guarner F., Reid G., Gibson G.R., Merenstein D.J., Pot B., Morelli L., Canani R.B., Flint H.J., Salminen S. (2014). Expert consensus document: The international scientific association for probiotics and prebiotics consensus statement on the scope and appropriate use of the term probiotic. Nat. Rev. Gastroenterol. Hepatol..

[4] Giri S.S., Sukumaran V., Sen S.S., Park S.C. (2018). Use of a potential probiotic, *Lactobacillus casei* L4, in the preparation of fermented coconut water beverage. Front. Microbiol..

[5] Konuray G., Erginkaya Z. (2018). Potential use of *Bacillus coagulans* in the food industry. Foods.

[6] Hemarajata P., Versalovic J. (2013). Effects of probiotics on gut microbiota: Mechanisms of intestinal immunomodulation and neuromodulation. Ther. Adv. Gastroenter..

[7] Remes-Troche J.M., Coss-Adame E., Valdovinos-Díaz M.A., Gómez-Escudero O., Icaza-Chávez M.E., Chávez-Barrera J.A., Zárate-Mondragón F., Velarde-Ruiz Velasco J.A., Aceves-Tavares G.R., Lira-Pedrín M.A. (2020). *Lactobacillus acidophilus* LB: A useful pharmabiotic for the treatment of digestive disorders. Ther. Adv. Gastroenter..

[8] Olveira G., González-Molero I. (2016). An update on probiotics, prebiotics and symbiotics in clinical nutrition. Endocrinol. Nutr..

[9] Li Y.T., Xu H., Ye J.Z., Wu W.R., Shi D., Fang D.Q., Liu Y., Li L.J. (2019). Efficacy of *Lactobacillus rhamnosus* GG in treatment of acute pediatric diarrhea: A systematic review with Meta-analysis. World J. Gastroenterol..

[10] Xue X., Yang X., Shi X., Deng Z. (2023). Efficacy of probiotics in pediatric atopic dermatitis: A systematic review and Meta-Analysis. Clin. Transl. Allergy.

[11] Climent E., Martinez-blanch J.F., Llobregat L., Ruzafa-costas B., Carrión-Gutiérrez M.Á., Ramírez-Boscá A., Prieto-Merino D., Genovés S., Codoñer F.M., Ramón D. (2021). Changes in gut microbiota correlates with response to treatment with probiotics in patients with atopic dermatitis. A Post Hoc analysis of a clinical trial. Microorganisms.

[12] Fijan S. (2014). Microorganisms with claimed probiotic properties: An overview of recent literature. Int. J. Environ. Res. Public Health.

[13] Tompkins T.A., Xu X., Ahmarani J. (2010). A comprehensive review of post-market clinical studies performed in adults with an Asian probiotic formulation. Benef. Microbes.

[14] Marseglia G.L., Tosca M., Cirillo I., Licari A., Leone M., Marseglia A., Castellazzi A.M., Ciprandi G. (2007). Efficacy of *Bacillus clausii* spores in the prevention of recurrent respiratory infections in children: A pilot study. Therap. Clin. Risk Manag..

[15] Qadri O.S., Yousuf B., Srivastava A.K. (2015). Fresh-cut fruits and vegetables: Critical factors influencing microbiology and novel approaches to prevent microbial risks—A review. Cogent Food Agric..

[16] Braïek O.B., Smaoui S. (2021). Chemistry, safety, and challenges of the use of organic acids and their derivative salts in meat preservation. J. Food Qual..

[17] Singh V.P. (2018). Recent approaches in food bio-preservation—A Review. Open Vet. J..

[18] Fliss I., Hammami R., Le Lay C., Lacroix C. (2010). Biological control of human digestive microbiota using antimicrobial cultures and bacteriocins. Protective Cultures, Antimicrobial Metabolites and Bacteriophages for Food and Beverage Biopreservation.

[19] Souza L.V., Martins E., Moreira I.M.F.B., de Carvalho A.F., Comi G. (2022). Strategies for the development of bioprotective cultures in food preservation. Int. J. Microbiol..

[20] De Vuyst L., Leroy F. (2007). Bacteriocins from lactic acid bacteria: Production, purification, and food applications. J. Mol. Microbiol. Biotechnol..

[21] Alvarez-Sieiro P., Montalbán-López M., Mu D., Kuipers O.P. (2016). Bacteriocins of lactic acid bacteria: Extending the family. Appl. Microbiol. Biotechnol..

[22] Abdhul K., Ganesh M., Shanmughapriya S., Vanithamani S., Kanagavel M., Anbarasu K., Natarajaseenivasan K. (2015). Bacteriocinogenic Potential of a probiotic strain *Bacillus coagulans* [BDU3] from Ngari. Int. J. Biol. Macromol..

[23] Abada E.A.E.M. (2008). Isolation and characterization of a antimicrobial compound from *Bacillus coagulans*. Anim. Cells Syst..

[24] Wang Y., Gu Z., Zhang S., Li P. (2023). complete genome sequencing revealed the potential application of a novel *Weizmannia coagulans* PL-W production with promising bacteriocins in food preservative. Foods.

[25] Wu M., Dong Q., Ma Y., Yang S., Zohaib Aslam M., Liu Y., Li Z. (2022). Potential antimicrobial activities of probiotics and their derivatives against *Listeria monocytogenes* in food field: A Review. Food Res. Int..

[26] Alegre I., Viñas I., Usall J., Anguera M., Abadias M. (2011). Microbiological and physicochemical quality of fresh-cut apple enriched with the probiotic strain *Lactobacillus rhamnosus* GG. Food Microbiol..

[27] Russo P., Peña N., de Chiara M.L.V., Amodio M.L., Colelli G., Spano G. (2015). Probiotic lactic acid bacteria for the production of multifunctional fresh-cut cantaloupe. Food Res. Int..

[28] De Senna A., Lathrop A. (2017). Antifungal Screening of bioprotective isolates against *Botrytis cinerea*, *Fusarium pallidoroseum* and *Fusarium moniliforme*. Fermentation.

[29] Ibrahim S.A., Yeboah P.J., Ayivi R.D., Eddin A.S., Wijemanna N.D., Paidari S., Bakhshayesh R.V. (2023). A review and comparative perspective on health benefits of probiotic and fermented foods. Int. J. Food Sci. Technol..

[30] Marco M.L., Sanders M.E., Gänzle M., Arrieta M.C., Cotter P.D., De Vuyst L., Hill C., Holzapfel W., Lebeer S., Merenstein D. (2021). The International Scientific Association for Probiotics and Prebiotics (ISAPP) Consensus statement on fermented foods. Nat. Rev. Gastroenterol. Hepatol..

[31] Mojikon F.D., Kasimin M.E., Molujin A.M., Gansau J.A., Jawan R. (2022). Probiotication of nutritious fruit and vegetable juices: An alternative to dairy-based probiotic functional products. Nutrients.

[32] Rasika D.M.D., Vidanarachchi J.K., Luiz S.F., Azeredo D.R.P., Cruz A.G., Ranadheera C.S. (2021). Probiotic delivery through non-dairy plant-based food matrices. Agriculture.

[33] Ruxton C.H.S., Myers M. (2021). Fruit Juices: Are they helpful or harmful? An evidence review. Nutrients.

[34] Maia M.S., Domingos M.M., de São José J.F.B. (2023). Viability of probiotic microorganisms and the effect of their addition to fruit and vegetable juices. Microorganisms.

[35] Tripathi M.K., Giri S.K. (2014). Probiotic functional foods: Survival of probiotics during processing and storage. J. Funct. Foods.

[36] Gibson G.R., Hutkins R., Sanders M.E., Prescott S.L., Reimer R.A., Salminen S.J., Scott K., Stanton C., Swanson K.S., Cani P.D. (2017). Expert consensus document: The International Scientific Association for Probiotics and Prebiotics (ISAPP) consensus statement on the definition and scope of prebiotics. Nat. Rev. Gastroenterol. Hepatol..

[37] Guerra L., Ureta M., Romanini D., Woitovich N., Gómez-Zavaglia A., Clementz A. (2023). Enzymatic synthesis of fructooligosaccharides: From carrot discards to prebiotic juice. Food Res. Int..

[38] Wei L., Sui H., Zhang J., Guo Z. (2021). Synthesis and antioxidant activity of the inulin derivative bearing 1,2,3-Triazole and diphenyl phosphate. Int. J. Biol. Macromol..

[39] Abadias M., Usall J., Anguera M., Solsona C., Viñas I. (2008). Microbiological Quality of fresh, minimally-processed fruit and vegetables, and sprouts from retail establishments. Int. J. Food Microbiol..

[40] Evers M.S., Roullier-Gall C., Morge C., Sparrow C., Gobert A., Vichi S., Alexandre H. (2023). Thiamine and biotin: Relevance in the production of volatile and non-volatile compounds during *Saccharomyces cerevisiae* alcoholic fermentation in synthetic grape must. Foods.

[41] Nicolau-Lapeña I., Abadias M., Bobo G., Lafarga T., Viñas I., Aguiló-Aguayo I. (2021). Antioxidant and Antimicrobial activities of ginseng extract, ferulic acid, and noni juice: Evaluation of their potential to be incorporated in food. J. Food Process. Preserv..

[42] Echeverría G., Graell J., López M.L., Lara I. (2004). Volatile Production, Quality and aroma-related enzyme activities during maturation of “Fuji” apples. Postharvest Biol. Technol..

[43] Min M., Bunt C.R., Mason S.L., Bennett G.N., Hussain M.A. (2017). Effect of non-dairy food matrices on the survival of probiotic bacteria during storage. Microorganisms.

[44] Chávez B.E., Ledeboer A.M. (2007). Drying of probiotics: Optimization of formulation and process to enhance storage survival. Dry. Technol..

[45] Senz M., van Lengerich B., Bader J., Stahl U. (2015). Control of cell morphology of probiotic *Lactobacillus acidophilus* for enhanced cell stability during industrial processing. Int. J. Food Microbiol..

[46] de Almeida Bianchini Campos R.C., Martins E.M.F., de Andrade Pires B., do Carmo Gouveia Peluzio M., da Rocha Campos A.N., Ramos A.M., de Castro Leite Júnior B.R., de Oliveira Martins A.D., da Silva R.R., Martins M.L. (2019). In vitro and in vivo resistance of *Lactobacillus rhamnosus* GG carried by a mixed pineapple (*Ananas comosus* L. Merril) and jussara (*Euterpe edulis Martius*) juice to the gastrointestinal tract. Food Res. Int..

[47] Pereira A.L.F., Almeida F.D.L., de Jesus A.L.T., da Costa J.M.C., Rodrigues S. (2013). Storage stability and acceptance of probiotic beverage from cashew apple juice. Food Bioprocess Technol..

[48] Fonteles T.V., Costa M.G.M., de Jesus A.L.T., Rodrigues S. (2012). Optimization of the fermentation of cantaloupe juice by *Lactobacillus casei* NRRL B-442. Food Bioprocess Technol..

[49] Konuray G., Erginkaya Z. (2020). Quality evaluation of probiotic pasta produced with *Bacillus coagulans* GBI-30. Innov. Food Sci. Emerg. Technol..

[50] Almada-Érix C.N., Almada C.N., Cabral L., Barros de Medeiros V.P., Roquetto A.R., Santos-Junior V.A., Fontes M., Gonçalves A.E.S.S., dos Santos A., Lollo P.C. (2021). Orange juice and yogurt carrying probiotic *Bacillus coagulans* GBI-30 6086: Impact of intake on Wistar male rats health parameters and gut bacterial diversity. Front. Microbiol..

[51] Rivas J.C., Cabral L.M.C., da Rocha-Leão M.H.M. (2021). Microencapsulation of guava pulp using prebiotic wall material. Braz. J. Food Technol..

[52] Pimentel T.C., Madrona G.S., Garcia S., Prudencio S.H. (2015). Probiotic viability, physicochemical characteristics and acceptability during refrigerated storage of clarified apple juice supplemented with *Lactobacillus paracasei* ssp. *paracasei* and oligofructose in different package type. LWT-Food Sci. Technol..

[53] Malik M., Bora J., Sharma V. (2019). Growth studies of potentially probiotic lactic acid bacteria (*Lactobacillus plantarum, Lactobacillus acidophilus* and *Lactobacillus casei*) in carrot and beetroot juice substrates. J. Food Process. Preserv..

[54] Costa M.G.M., Fonteles T.V., De Jesus A.L.T., Rodrigues S. (2013). Sonicated pineapple juice as substrate for *L. casei* cultivation for probiotic beverage development: Process optimisation and product stability. Food Chem..

[55] Yang J., Sun Y., Gao T., Wu Y., Sun H., Zhu Q., Liu C., Zhou C., Han Y., Tao Y. (2022). Fermentation and storage characteristics of “Fuji” apple juice using *Lactobacillus acidophilus*, *Lactobacillus casei* and *Lactobacillus plantarum*: Microbial growth, metabolism of bioactives and *in vitro* bioactivities. Front. Nutr..

[56] Lavrentev F.V., Ashikhmina M.S., Ulasevich S.A., Morozova O.V., Orlova O.Y., Skorb E.V., Iakovchenko N.V. (2021). Perspectives of *Bacillus coagulans* MTCC 5856 in the production of fermented dairy products. LWT-Food Sci. Technol..

[57] Majeed M., Nagabhushanam K., Arumugam S., Natarajan S., Majeed S., Pande A., Beede K., Ali F. (2018). Cranberry seed fibre: A promising prebiotic fibre and its fermentation by the probiotic *Bacillus coagulans* MTCC 5856. Int. J. Food Sci. Technol..

[58] Maftei N.M., Iancu A.V., Goroftei Bogdan R.E., Gurau T.V., Ramos-Villarroel A., Pelin A.M. (2023). A novel symbiotic beverage based on sea buckthorn, soy milk and inulin: Production, characterization, probiotic viability, and sensory acceptance. Microorganisms.

[59] Nancib A., Nancib N., Meziane-Cherif D., Boubendir A., Fick M., Boudrant J. (2005). Joint effect of nitrogen sources and B vitamin supplementation of date juice on lactic acid production by *Lactobacillus casei* subsp. rhamnosus. Bioresour. Technol..

[60] Ohara H., Yahata M. (1996). L-Lactic acid production by *Bacillus* sp. in anaerobic and aerobic culture. J. Ferment. Bioeng..

[61] Baráth Á., Halász A., Németh E., Zalán Z. (2004). Selection of LAB strains for fermented red beet juice production. Eur. Food Res. Technol..

[62] Budak N.H., Özdemir N., Gökırmaklı Ç. (2022). The changes of physicochemical properties, antioxidants, organic, and key volatile compounds associated with the flavor of peach (*Prunus Cerasus* L. Batsch) vinegar during the fermentation process. J. Food Biochem..

[63] Abdel-Daim A., Hassouna N., Hafez M., Ashor M.S.A., Aboulwafa M.M. (2013). Antagonistic activity of *Lactobacillus* isolates against *Salmonella typhi* in vitro. Biomed. Res. Int..

[64] Divyashree S., Anjali P.G., Somashekaraiah R., Sreenivasa M.Y. (2021). Probiotic properties of *Lactobacillus casei*–MYSRD 108 and *Lactobacillus plantarum*-MYSRD 71 with potential antimicrobial activity against *Salmonella paratyphi*. Biotechnol. Rep..

[65] Reuben R.C., Elghandour M.M.M.Y., Alqaisi O., Cone J.W., Márquez O., Salem A.Z.M. (2022). Influence of microbial probiotics on ruminant health and nutrition: Sources, mode of action and implications. J. Sci. Food Agric..

[66] Servin A.L. (2004). Antagonistic activities of *Lactobacilli* and *Bifidobacteria* against microbial pathogens. FEMS Microbiol. Rev..

[67] Woo J., Ahn J. (2013). Probiotic-Mediated Competition, Exclusion and displacement in biofilm formation by food-borne pathogens. Lett. Appl. Microbiol..

[68] De Waard R., Garssen J., Bokken G.C.A.M., Vos J.G. (2002). Antagonistic activity of *Lactobacillus casei* strain Shirota against gastrointestinal *Listeria monocytogenes* infection in rats. Int. J. Food Microbiol..

[69] Abou Elez R.M.M., Elsohaby I., Al-Mohammadi A.R., Seliem M., Tahoun A.B.M.B., Abousaty A.I., Algendy R.M., Mohamed E.A.A., El-Gazzar N. (2023). Antibacterial and anti-biofilm activities of probiotic *Lactobacillus plantarum* against *Listeria monocytogenes* isolated from milk, chicken and pregnant women. Front. Microbiol..

[70] Zhang J., Gu S., Zhang T., Wu Y., Ma J., Zhao L., Li X., Zhang J. (2022). Characterization and antibacterial modes of action of bacteriocins from *Bacillus coagulans* CGMCC 9951 against *Listeria monocytogenes*. LWT-Food Sci. Technol..

[71] Rugji J., Dinçoğlu A.H. (2022). Biocontrol of *Listeria monocytogenes* by *Bacillus coagulans* GBI-30, 6086 in a synbiotic white brined cheese: An *in vitro* model study. LWT-Food Sci. Technol..

[72] Huang J., Luo Y., Zhou B., Zheng J., Nou X. (2019). Growth and Survival of *Salmonella enterica* and *Listeria monocytogenes* on fresh-cut produce and their juice extracts: Impacts and interactions of food matrices and temperature abuse conditions. Food Control.

[73] Leneveu-Jenvrin C., Quentin B., Assemat S., Remize F. (2020). Maintaining physicochemical, microbiological, and sensory quality of pineapple juice (*Ananas comosus*, Var. ’Queen Victoria’) through mild heat treatment. Processes.

[74] Gouvinhas I., Santos R.A., Queiroz M., Leal C., Saavedra M.J., Domínguez-Perles R., Rodrigues M., Barros A.I.R.N.A. (2018). Monitoring the antioxidant and antimicrobial power of grape (*Vitis vinifera* L.) stems phenolics over long-term storage. Ind. Crops Prod..

[75] Yang E., Fan L., Jiang Y., Doucette C., Fillmore S. (2012). Antimicrobial activity of bacteriocin-producing lactic acid bacteria isolated from cheeses and yogurts. AMB Express.

[76] Moradi M., Mardani K., Tajik H. (2019). Characterization and application of postbiotics of *Lactobacillus* spp. on *Listeria monocytogenes in vitro* and in food Models. LWT-Food Sci. Technol..

[77] Bagheri L., Khlifi M., Maherani B., Salmieri S., Lacroix M. (2020). Thermosensitization enhancement of *A. niger, S. cerevisiae* and *L. fructivorans* using combination of mild heat treatment with nanoemulsion-based mediterranean formulation to fabricate wholesome orange juice. LWT-Food Sci. Technol..

[78] Gündüz G.T., Korkmaz A., Solak E., Sözbir H.D. (2019). Antimicrobial, antioxidant activities and total phenolic contents of the traditional turkish beverages produced by using grapes. Turkish JAF Sci. Tech..

[79] Makarewicz M., Drożdż I., Tarko T., Duda-Chodak A. (2021). the interactions between polyphenols and microorganisms, especially gut microbiota. Antioxidants.

[80] Panigrahi C., Mishra H.N., De S. (2021). Modelling the inactivation kinetics of *Leuconostoc mesenteroides*, *Saccharomyces cerevisiae* and total coliforms during ozone treatment of sugarcane juice. LWT-Food Sci. Technol..

[81] Alexopoulos A., Plessas S., Ceciu S., Lazar V., Mantzourani I., Voidarou C., Stavropoulou E., Bezirtzoglou E. (2013). Evaluation of Ozone efficacy on the reduction of microbial population of fresh cut lettuce (*Lactuca sativa*) and green bell pepper (*Capsicum annuum*). Food Control.

[82] Cheirsilp B., Shoji H., Shimizu H., Shioya S. (2003). Interactions between *Lactobacillus kefiranofaciens* and *Saccharomyces cerevisiae* in mixed culture for kefiran production. J. Biosci. Bioeng..

[83] Paramithiotis S., Gioulatos S., Tsakalidou E., Kalantzopoulos G. (2006). Interactions between *Saccharomyces cerevisiae* and Lactic Acid Bacteria in sourdough. Process Biochem..

[84] Tiwari B.K., Rice R.G., O’Donnell C., Tiwari B.K., Cullen P.J., Rice R.G. (2012). Regulatory and legislative issues. Ozone in Food Processing.

[85] Bassi A.P.G., Meneguello L., Paraluppi A.L., Sanches B.C.P., Ceccato-Antonini S.R. (2018). Interaction of *Saccharomyces cerevisiae–Lactobacillus fermentum–Dekkera bruxellensis* and feedstock on fuel ethanol fermentation. Antonie van Leeuwenhoek.

[86] Carvalho R.S., Cruz I.A., Américo-Pinheiro J.H.P., Soriano R.N., de Souza R.L., Bilal M., Iqbal H.M.N., Bharagava R.N., Romanholo Ferreira L.F. (2020). Interaction between *Saccharomyces cerevisiae* and *Lactobacillus fermentum* during co-culture fermentation. Biocatal. Agric. Biotechnol..

[87] Yüksekdaǧ Z.N., Beyath Y., Aslim B. (2004). Metabolic Activities of *Lactobacillus* spp. strains isolated from kefir. Mol. Nutr. Food Res..

[88] Li E., Mira De Orduña R. (2011). Evaluation of the acetaldehyde production and degradation potential of 26 enological *Saccharomyces* and non-*Saccharomyces* yeast strains in a resting cell model system. J. Ind. Microbiol. Biotechnol..

[89] Lachenmeier D.W., Kanteres F., Rehm J. (2009). Carcinogenicity of acetaldehyde in alcoholic beverages: Risk assessment outside ethanol metabolism. Addiction.

[90] Attfield P.V. (2023). Crucial Aspects of metabolism and cell biology relating to industrial production and processing of *Saccharomyces* Biomass. Crit. Rev. Biotechnol..

